# Lumenato protects normal human dermal fibroblasts from neutrophil-induced collagen-3 damage in co-cultures

**DOI:** 10.1371/journal.pone.0248183

**Published:** 2021-03-17

**Authors:** Yulia Solomonov, Nurit Hadad, Oleg Pikovsky, Rachel Levy

**Affiliations:** 1 Immunology and Infectious Diseases Laboratory, Department of Clinical Biochemistry and Pharmacology, Faculty of Health Sciences, Ben-Gurion University of the Negev, Beer-Sheva, Israel; 2 Blood Bank and Pheresis Unit, Soroka University Medical Center, Beer-Sheva, Israel; B. S. Abdur Rahman Crescent Institute of Science and Technology, INDIA

## Abstract

Collagen is the major structural protein in the extracellular matrix of skin produced by fibroblasts. UV exposure results in infiltration of neutrophils within the epidermis and dermis, inducing collagen damage and contributing to the process of photo-aging. Collagen-3 is an integral structural component with collagen-1, and is an important regulator of collagen-1 fibrillogenesis. Addition of neutrophils activated with TNFα to normal human dermal fibroblast cultures, but not their supernatant, caused significant collagen-3 damage. To study whether Lumenato can protect from collagen-3 damage, it was added to co-cultures of Normal human dermal fibroblasts and neutrophils activated with TNFα. Lumenato prevented collagen-3 damage induced by activated neutrophils in a dose-dependent manner in the co-cultures. Lumenato also induced a low rate of collagen-3 synthesis in a dose-dependent manner detected by pro-collagen-3 secretion, but did not affect fibroblast cell number. Although Lumenato inhibited MMP-8, MMP-9, and elastase secreted from neutrophils, its main effect was in inhibiting both NADPH oxidase-producing superoxides and MPO activity-producing halides in a dose-dependent manner that correlated with protection from collagen-3 damage. In conclusion, the results suggest that Lumenato induces low levels of collagen-3 that may contribute for skin health and is very effective in defending the co-cultures from collagen-3 damage by inhibiting free radicals secreted from neutrophils, thus, indicating Lumenato's possible potential for skin protection.

## 1. Introduction

Dermal collagen represents the most abundant extracellular matrix (ECM) protein and constitutes the bulk of human skin [1]. The major components of the dermis are fibroblasts that produce, organize, and maintain collagen and elastic fibers [2]. Type-3 collagen is a member of the fibrillar collagen family and is co-localized with the most abundant member of the family, type-I collagen, in the skin [3, 4]. In addition to being an integral structural component, it is also an important regulator of type-I collagen fibrillogenesis, determining the diameter of the fibrils. Collagen-I fibrils in skin of mice with mutant COL3A1 were disorganized and highly variable in diameter compared with those of wild-type mice [5]. Patients with type IV Ehlers–Danlos syndrome, a genetic disorder associated with fragile skin and blood vessels, carry mutations in the COL3A1 gene coding for type-3 pro-collagen [6, 7]. Synthesis of collagen-3 is reduced in aged and photodamaged skin, possibly resulting in altered organization of fibrillar collagen and, thus, contributing to the wrinkled appearance of skin [8, 9]. Collagen-3 is the first to emerge and acts as a bridge to the wound, after which type-1 collagen appears in conjunction with type-3 during tissue reformation to build a solid bracket and facilitate wound healing [10].

Inflammation and accumulation of reactive oxygen species are now believed to be the causative factors in both types of skin aging: intrinsic (or chronological) and extrinsic UV-induced aging (or photoaging) [11–13]. Several global gene expression profiling studies have linked the immune system and inflammation genes with photoaging, regardless of ethnic type [14–16]. UV induces an array of events that can lead to inflammation: release of inflammatory cytokines by epidermal keratinocytes and fibroblasts such as tumor necrosis factor-alpha (TNFα) [17], ROS generation [18], production of inflammatory mediators [19], peroxidation of the membrane lipids [20], and skin cell death [21]. Neutrophils have been reported to infiltrate the skin within the epidermis and dermis following exposure to natural sunlight to erythemogenic doses of UVB and solar simulating radiation (SSR), infrared radiation, and heat [22–25]. These neutrophils are packed with potent proteolytic enzymes for clearance of UV-induced apoptotic cells and for killing skin cells with oxidized surface lipids, but they are also capable of degrading collagen and elastic fibers [26, 27]. Furthermore, it was reported that infiltrating neutrophils, rather than keratinocytes and fibroblasts, were the major source of proteolytic enzymes following exposure to erythemogenic doses of SSR, and thus may be the key players in photoaging [24, 28]. Lumenato, a golden tomato extract, is composed mainly of the colorless phytoene and phytofluene, light yellow zeta carotene, tocopherols, and phytosterols. In the present study, we examined the role of Lumenato in preventing fibroblast collagen-3 degradation induced by stimulated neutrophils using a co-culture of neutrophils and normal human dermal fibroblasts of adult donors.

## 2. Materials and methods

### 2.1 Materials

Lumenato, composed of 0.55% lycopene, 24.06% phytoene, 6.75% phytofluene, 11.27% tocopherols, 0.61% b-carotene, 3.15% phytosterols, 7.48% zeta-carotene, gamma carotene 0.16% and tomato seed oil was supplied by LycoRed Natural Products Industries Ltd. (Beer-Sheva, Israel).

### 2.2 Preparation of human neutrophils

Forty ml blood with neutrophil count between 3-7x10^6^/ml was drawn from healthy volunteers with their written consent. The study was approved by the Helsinki Committee of the Soroka Medical Center (No. 0370-16-SOR). Neutrophils at 95% purity were obtained by Ficoll-Hypaque centrifugation, dextran sedimentation, and hypotonic lysis of erythrocytes within 1h of blood drawn, as done before [29]. Cells were counted and their viability was determined by trypan blue exclusion.

### 2.3 Cell culture

Normal human dermal fibroblasts (NHDF) of adult donors (Promocell, Heidelberg, Germany), were cultured in and supplemented with Fibroblast Growth Medium-2 (Promocell) (final supplemental concentration in the medium: fetal calf serum 0.02ml/ml, basic fibroblast recombinant human growth factor 1mg/ml, and recombinant human insulin 5μg/ml), 2 mM L-glutamine, 100 U/ml penicillin, and 100 μg/ml streptomycin (Beit-Haemek, Israel). The cells were maintained at 37°C in a humidified atmosphere containing 5% CO_2_. When fibroblasts reached more than 80% confluence, the cells were seeded in 24-well plates.

### 2.4 Cell survival

Cell viability was assessed by cell count using trypan blue exclusion or the colorimetric MTT ([*3*–4, *5*-*dimethyl*thiazol-2-yl]-2,*5*-diphenyl-tetrazolium) metabolic activity assay, as done before [30]. For MTT measurement, cells were cultured in 96-well plates. The MTT was dissolved in medium (5 mg/ml) and added to each sample in an amount equal to 10% of the culture medium volume. After incubation for 30 min, the formazan crystals were dissolved in 100 mM HCl and 10% Triton X-100, all in isopropanol in an equal volume to the culture medium. The medium only served as a background. Absorbance intensity was measured by a Versamax Microplate Reader (Molecular Devices, Menlo Park, CA, USA) at 570 nm with a reference wavelength of 690 nm.

### 2.5 Immunofluorescence analysis

For immunofluorescence detection, fibroblasts or co-cultures of fibroblasts and neutrophils were fixed with methanol at -20°C for 3 min followed by a wash in PBS. Fixed fibroblasts were incubated with anti-collagen-3 antibodies (Santa Cruz) 1:50 in 5% BSA/PBS for 90 min at room temperature. The cells were washed three times in PBS and incubated with Cy3 anti-mouse, (1:100 in 5% BSA/PBS; Jackson ImmunoResearch Laboratories, Inc., PA, USA) for 60 min at room temperature. The cells were washed three times in PBS, and the nuclei were stained with DAPI. Then a final wash was performed, and the cells were analyzed by fluorescence microscopy (Olympus, BX60, Hamburg, Germany). Fluorescence intensity was determined for collagen-3 using a CellProfiler Program. To support and confirm our immunofluorescence staining results we also used Pannoramic MIDI II with 3DHISTECH software (3DHISTECH Ltd. Budapest Hungary) for scanning the whole cover slip as shown in the example (S1 Fig).

### 2.6 Superoxide generation

**A**. Cytochrome C reduction—The production of the superoxide anion by neutrophils was measured as the superoxide dismutase inhibitable reduction of ferricytochrome c by the microtiter plate technique, as previously described [29]. Cells (2.5x10^5^/well) were suspended in 100 μl Hanks' Balanced Salt Solution (HBSS) containing ferricytochrome c (150 mM). The reduction of acetyl ferricytochrome c was followed by the change of absorbance at 550 nm at 2-min intervals on a Versamax Microplate Reader (Molecular Devices, Menlo Park, CA). The maximal rates of superoxide generation were determined and expressed as nmoles O_2_^-^/10^6^ cells/min using the extinction coefficient E_550_ = 21 mM^-1^cm^-1^. **B**. Horseradish peroxidase (HRP)-dependent oxidation of the highly sensitive fluorescent biosensor Amplex Red [31]. The oxidation of Amplex Red occurs outside the cells by HRP, which traps H_2_O_2_ as soon as it is generated by spontaneous dismutation of O_2_^-^, the first product of NADPH oxidase. Resting and activated neutrophils (2x10^4^/well) were suspended in KRPG buffer (phosphate buffer, 145 mM NaCl, 4.86 mM KCl, 1.22 mM MgSO_4_, 5.5 mM D-glucose, 0.54 mM CaCl_2_, pH 7.35) containing HRP (0.1 unit/ml) and Amplex Red (50 uM). Fluorescence was recorded using a microplate reader with 535nm-excitation and 595nm-emission wavelengths. Background fluorescence was measured in the absence of neutrophil cells.

### 2.7 Myeloperoxidase (MPO) activity

100 μl of 37°C O-dianisidine hydrochloride solution (1 mg O-dianisidine, 10ml phosphate buffer pH 6.0 + 0.0015% H_2_O_2_) was added to 100 μl supernatant in a 96-well plate immediately before the optical density was followed by the change of absorbance at 450 nm at 2-min intervals on a Thermomax Microplate Reader (Molecular Devices, Menlo Park, CA).

### 2.8 MMP-9

Human MMP-9 concentrations in cell culture supernatants were quantified by ELISA kits (R&D Systems Minneapolis, MN, USA).

### 2.9 MMP-8 (collagenase)

Human MMP-8 concentrations in cell-culture supernatants were quantified by ELISA kits (OriGene, Technologies Inc, Rockville, MD, USA).

### 2.10 Elastase activity

To 100 μl supernatant, N–methoxysuccinyl-ala-ala-pro-Val-P-nitroanilide was added at a final concentration of 0.5 mM and incubated overnight at 37°C. The absorbance at 405 nm was measured on a Thermomax Microplate Reader (Molecular Devices, Men Park, CA).

### 2.11 Pro-collagen-3

Human pro-collagen-3 concentrations in cell-culture supernatants were quantified by a Human PⅢNP (N-terminal pro-collagen Ⅲ propeptide) ELISA kit (Elabscience Biotechnology Inc. Houston, Texas, USA). Since the concentration of pro-collagen-3 in the supernatants of the fibroblasts was very low, the supernatants were concentrated by evaporation.

### 2.12 Collagen-3

Human collagen-3 concentrations in cell-culture lysates and supernatants were quantified by a human collagen, type III, alpha 1 (COL3A1) ELISA kit (Cusabio Technology Inc., Houston, Texas, USA).

### 2.13 Statistical analysis

Significant differences between the parameters evaluated were determined by ANOVA using GraphPad Prism 5 (GraphPad Software Inc., San Diego, CA, USA), followed by multiple comparisons using Bonferroni post hoc correction.

## 3. Results

### 3.1 The effect of Lumenato on activated human neutrophils

**Fig 1:**
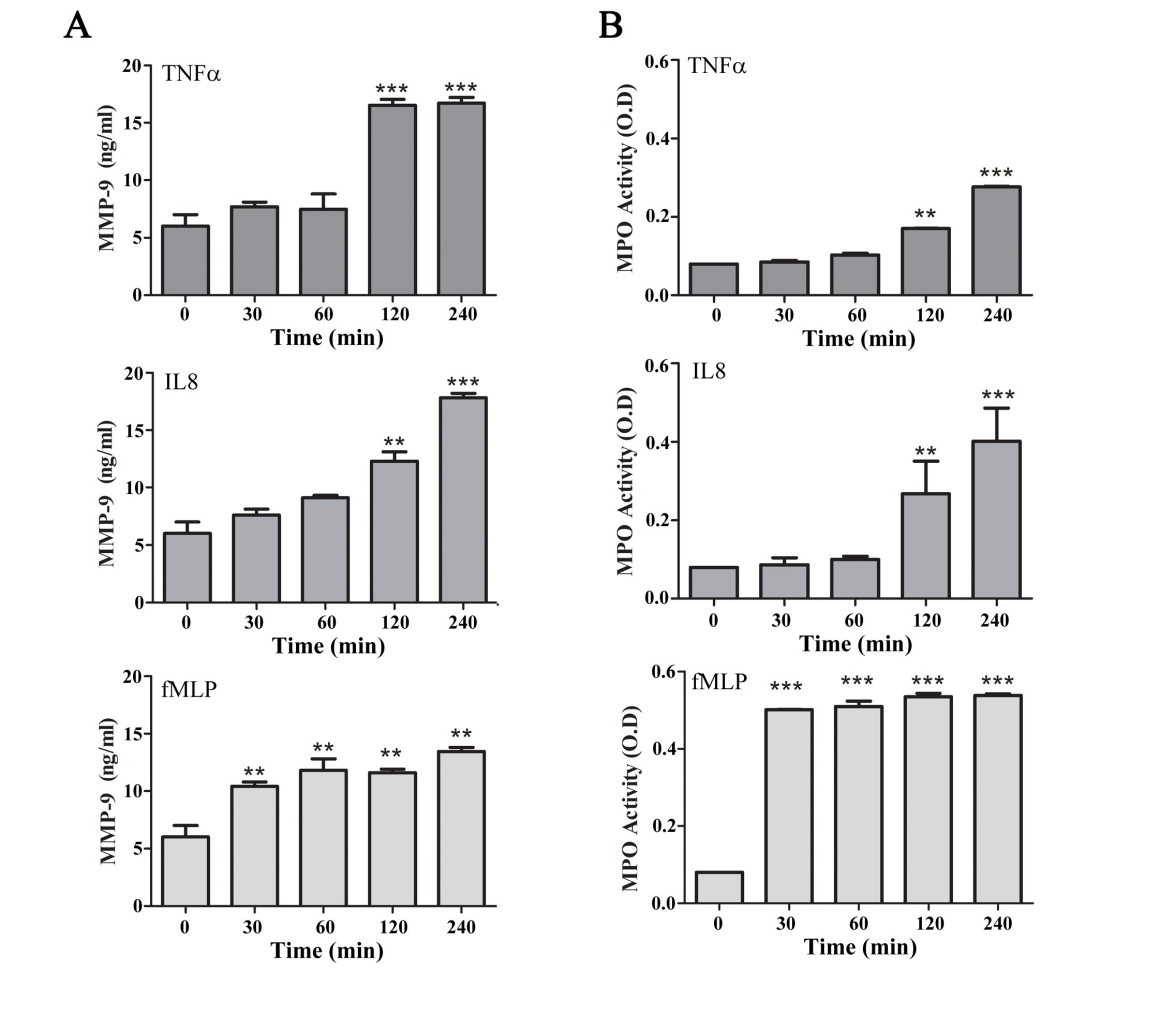
MMP-9 and of MPO secretion from activated human neutrophils. The kinetics of MMP-9 and of MPO secretion detected by their activity from human neutrophils stimulated by 100 ng/ml TNFα, 100 ng/ml IL-8, or 10^−5^ M fMLP. Average ± SEM of five independent experiments each in duplicates. Significance: ** p<0.01, *** p<0.001.

In order to study the effect of Lumenato on the release of enzymes that may have the potential to cause damage to collagen-3, we first determined the kinetics of the release of such enzymes from activated neutrophils. Neutrophils (5X10^6^/ml) were activated by 100 ng/ml TNFα or 100 ng/ml IL-8 that were shown to be released from the skin cells during exposure to UV [17]. Neutrophils were also activated with 10^-5^M fMLP that is released from bacteria during infection for comparison. As shown in Fig 1A, addition of TNFα to human neutrophils caused a significant (p<0.001) release of MMP-9 at 2h of activation (16.52±0.5 ng/ml), a level which was sustained at 4h. Addition of IL-8 caused a release of MMP-9 in a dose-dependent manner during 4h of activation. A significant p<0.01 release of MMP-9 was detected at 2h of activation with IL-8 (9.12±0.2 ng/ml) that was further significantly (p<0.001) increased at 4h to 17.86±0.4 ng/ml. fMLP induced a rapid and significant (p<0.01) release of MMP-9 at 30 min of activation (10.41±0.4 ng/ml), which was gradually increased during 4h of activation to 13.45±0.3 ng/ml. As shown in Fig 1B, addition of either TNFα or IL-8 caused a significant (p<0.01) release of MPO at 2h of activation, which was further significantly increased (p<0.001) at 4h to 0.28±0.01 OD and to 0.4±0.08 OD, respectively. fMLP induced a rapid and significant (p<0.001) release of MPO at 30 min to 0.54±0.01 OD which was sustained at this level for 4h. Activation of neutrophils overnight did not augment the release of MMP-9 or MPO compared with 4h (not shown).

**Fig 2:**
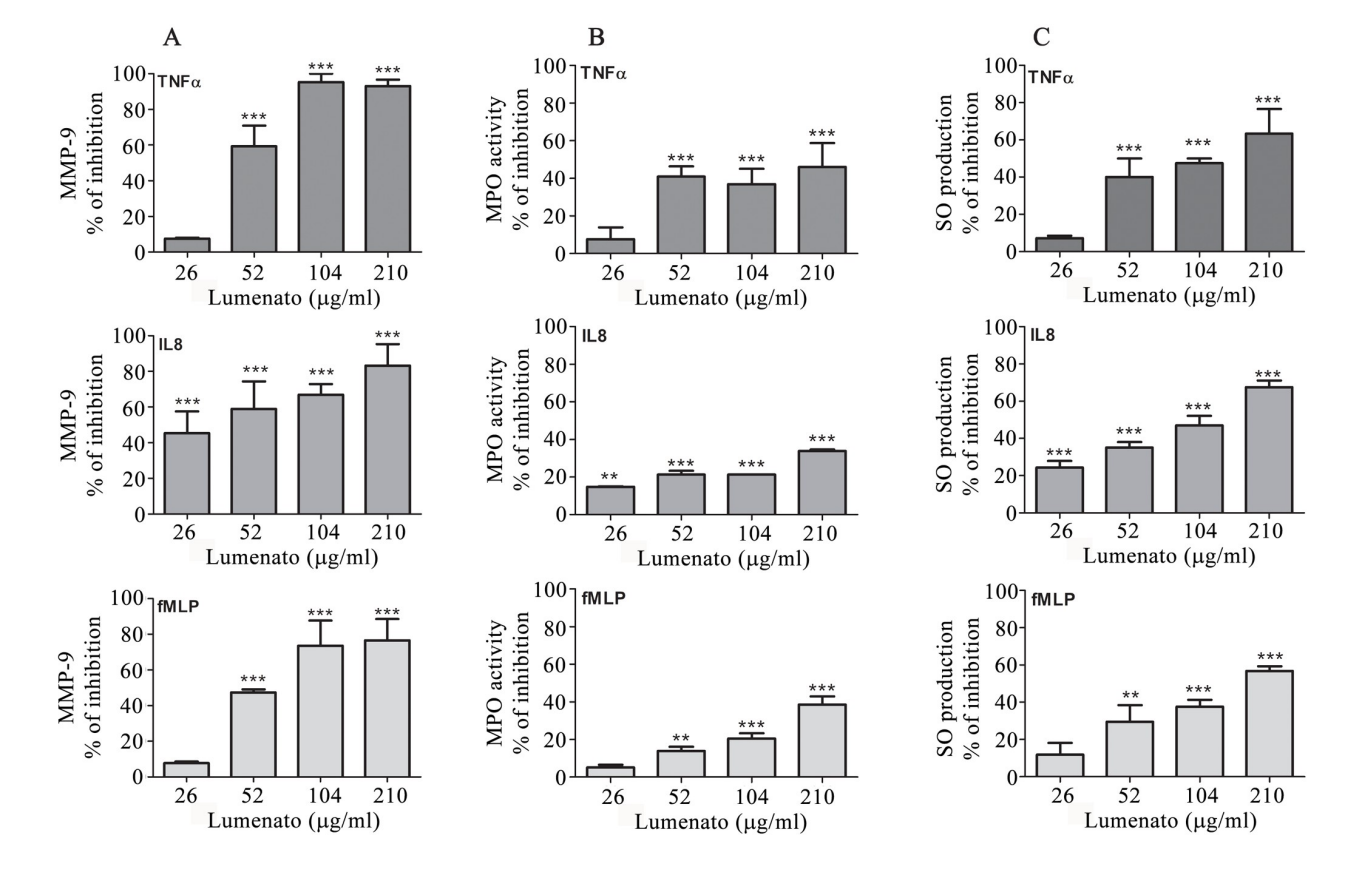
Inhibition of MMP-9, MPO secretion, and superoxide production by Lumenato. Dose-dependent inhibition of MMP-9, MPO secretion, and superoxide (SO) production by Lumenato from human neutrophils stimulated by different agonists. Neutrophils were incubated with Lumenato 15 min before addition of 100 ng/ml TNFα or 100 ng/ml IL-8 for 2h. Superoxide production was measured by cytochrome C reduction immediately after addition of either of the stimuli. Average ± SEM of three independent experiments each in duplicates. Significance: ** p<0.01, *** p<0.001.

To study the effect of Lumenato on the release of MMP-9 or MPO, it was added to neutrophils for 15 min at 37°C before activation by TNFα or IL8 for 4h and by fMLP for 30 min. As shown in Fig 2A, addition of Lumenato in a range of 26–210 ug/ml inhibited the release of MMP-9 from neutrophils stimulated by either TNFα, IL-8, or fMLP in a dose-dependent manner. Maximal inhibition of MMP9 was 95.4±4.6%, 83.19±12.0% or 76.6±12.0% from neutrophils stimulated by either TNFα, IL-8, or fMLP, respectively. Similarly, Lumenato caused a dose-dependent inhibition of MPO with maximal inhibition of 46.0±6.7%, 33.86±0.7%, or 38.5±4.5% from neutrophils stimulated by TNFα, IL-8, or fMLP, respectively (Fig 2B). Next, we analyzed the effect of Lumenato on superoxides released from neutrophils by cytochrome C reduction, immediately after the addition of each of the stimuli since superoxides are not stable. As shown in Fig 2C, addition of Lumenato for 15 min before a 20-min stimulation with TNFα caused a dose-dependent inhibition of superoxide production, reaching maximal inhibition of 63.27±13% when stimulated by TNFα, 67.55±3.4% when stimulated by IL-8, and 56.81±2.4% when stimulated by fMLP.

### 3.2 The effect of activated neutrophils on fibroblast collagen-3 damage

**Fig 3:**
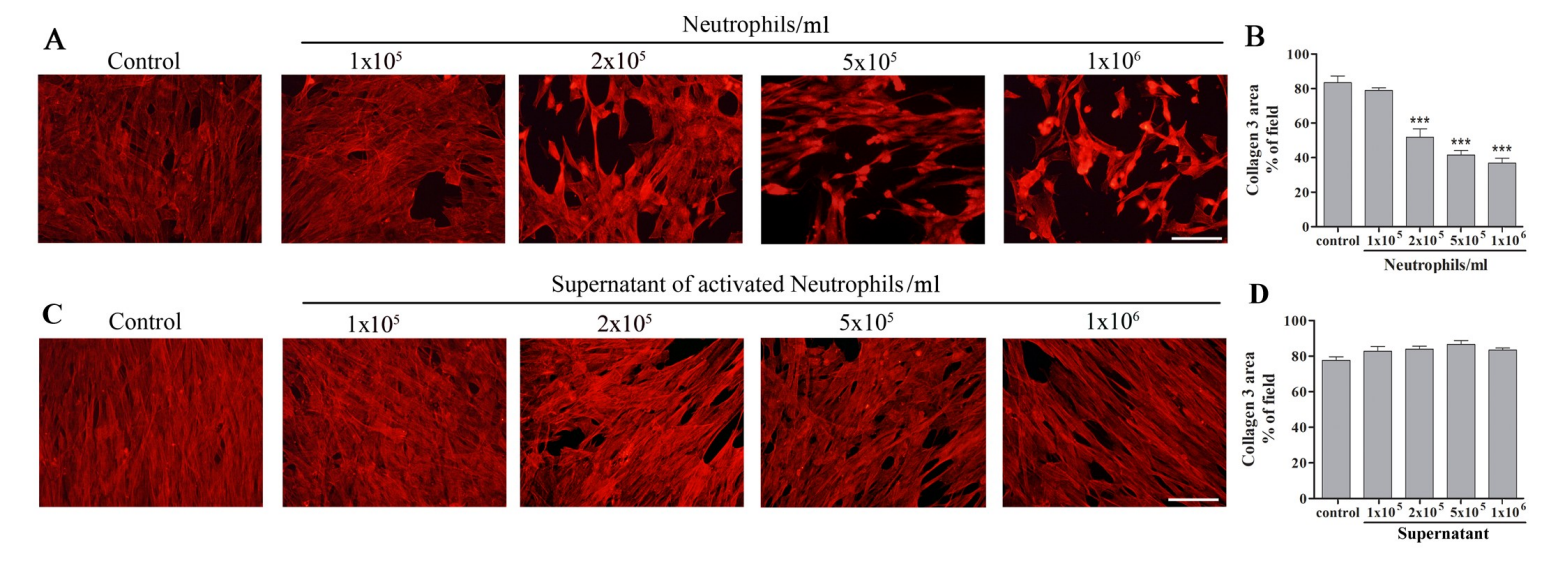
Damage of fibroblast collagen-3 induced by activated neutrophils. Addition of activated neutrophils (A), but not supernatant of activated neutrophils (C) caused damage of fibroblast collagen-3 in a dose-dependent manner. A representative experiment out to three independent experiments is presented, Scale bars 100 μm. The bar graph (B and D) presents the densitometry of the immunofluorescence staining of collagen-3 in A and B, respectively. Shown are the means± SEM of three different experiments. In each experiment, five fields of each treatment were scanned. Significance: *** p<0.001.

In order to study the effect of Lumenato on the damage to collagen-3 induced by activated neutrophils, we defined the optimal conditions of co-cultures of fibroblasts and neutrophils. 1X10^5^/ml fibroblasts were plated overnight to obtain confluent cultures (S2 Fig). Activated neutrophils by 100 ng/ml TNFα or the supernatant of activated neutrophils for 2 h at 37°C in the range of 10^5^–10^6^ neutrophils/ml were added to the fibroblasts for overnight. Collagen-3 was specifically detected by immuno-fluorescence analysis (S3 Fig) that detects external and internal collagen-3. As shown in Fig 3A addition of 1X10^5^ neutrophils/ml activated with TNFαdid not affect collagen-3 expression. Addition of the activated neutrophils in the range of 2X10^5^ /ml– 1X10^6^/ml caused collagen-3 damage in a dose dependent manner. Addition of 2X10^5^ activated neutrophils/ml with TNFαcaused significant collagen-3 damage of 32.8±5.8%, as shown by immunofluorescence staining of collagen-3 (Fig 3B), and this neutrophil concentration was used to study the effect of Lumenato on collagen-3 damage induced by neutrophils. This concentration of neutrophils was lower than that used in Figs 1 and 2 to study the effect of Lumenato on neutrophils. Addition of the supernatants of the activated neutrophil for overnight did not cause any collagen-3 damage (Fig 3C and 3D), indicating the necessity of the neutrophils for the damage effect. Similarly, addition of TNFα to control fibroblasts overnight did not affect collagen-3 expression as shown in S4 Fig.

### 3.3. The protective effect of Lumenato on collagen damage

**Fig 4:**
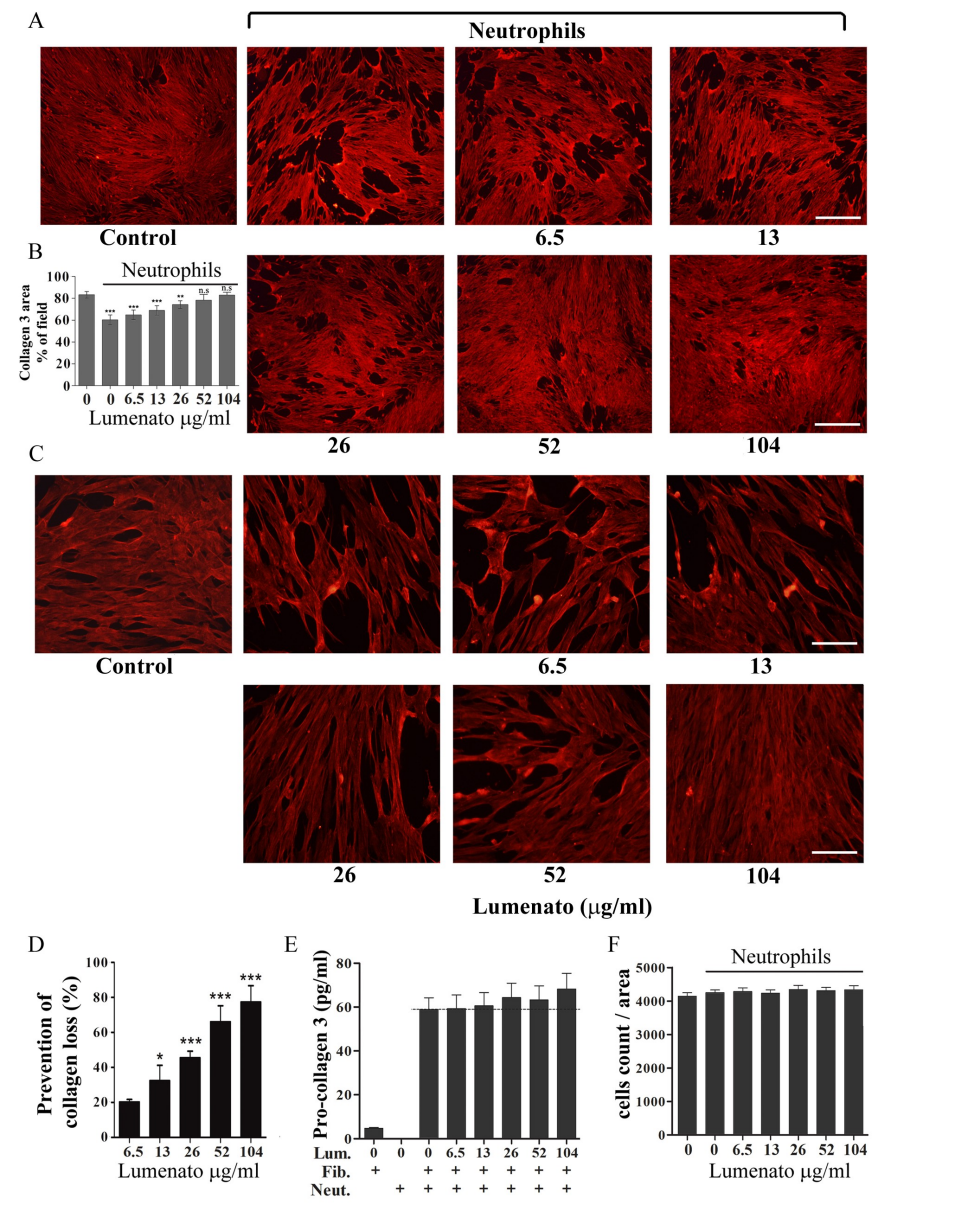
Prevention of collagen-3 damage by Lumenato. Addition of Lumenato prevented in a dose-dependent manner collagen-3 damage induced by neutrophils stimulated with 100 ng/ml TNFα. 1X10^5^/ml fibroblasts were plated for 24h to obtain confluent cultures before the addition of neutrophils. Neutrophils 5X10^5^/ml in growth medium were incubated with Lumenato for 15 min at 37°C before activation with 100 ng/ml TNFα for 15 min, and were then replaced with the medium of the fibroblast cultures and incubated overnight. A and C represent immunofluorescence staining of collagen-3, Scale bars 100 μm and 500 μm, respectively. B. The bar graph presents the densitometry of the immunofluorescence staining of collagen-3 in A. Shown are the means± SEM of three different experiments. In each experiment, ten fields of each treatment were scanned. D. The bar graph presents the calculated % of protection of collagen-3 damage after scanning the densitometry of the immunofluorescence staining of collagen-3. Shown are the mean ± SEM of five different experiments. In each experiment, ten fields of each treatment were scanned. E. Secreted pro-collagen-3 to the supernatant of the co-cultures. Shown are the mean ± SEM of three different experiments each in triplicates. The horizontal dashed line indicates the effect of Lumenato without the effect of neutrophils. Lum- Lumenato (ug/ml), Fib- fibroblasts, Neu–neutrophils. F. Cell count by DAPI staining in the cultures described in A and B. Significance: *p<0.05, ** p<0.01, *** p<0.001, n.s. -non significant.

To study whether Lumenato has a protective effect on fibroblast collagen-3 damage induced by activated neutrophils, 2X10^5^/ml neutrophils were incubated with Lumenato for 15 min at 37°C before their activation with 100 ng/ml TNFα and then added to the fibroblast cultures for overnight. As shown by immunofluorescence staining of collagen-3 (Fig 4A and 4C), addition of neutrophils caused collagen-3 damage (28.6±3.5%) that was inhibited in dose-dependent manner in the presence of Lumenato in the range of 6.5–104 ug/ml. The bar graph (Fig 4D) presents the calculated protection of collagen-3 in the cultures deduced from the densitometry. The maximal prevention of collagen-3 damage was reached at 104 ug/ml Lumenato (77.4±8.3% protection), and higher concentrations of Lumenato caused a similar protective effect (not shown). The concentration of collagen-3 in fibroblast lysate was determined and was found to be 0.45±0.05 ug/ml. To determine whether Lumenato contributes to the reduced collagne-3 damage by inducing collagen-3 synthesis, pro-collagen-3 (which is commonly used to determine collagen synthesis) was assayed in the supernatant of the co-cultures. As shown in Fig 4E, addition of stimulated neutrophils caused a significant elevation of pro-collagen-3 in the supernatant probably due to turnover in accordance with the literature [32] from 4.77±04 pg/ml in fibroblasts to 58.8±5.35 pg/ml in the co-cultures. However, the synthesis of pro-collagen-3 by neutrophils was negligible in comparison with the damage in their presence that reached 28.6%. The presence of Lumenato in the co-cultures caused a low dose-dependent non-significant increase in the secreted pro-collagen-3 to 68.21±7.1 pg/ml in the presence of 104 ug/ml Lumenato (Fig 4E). Subtracting the level of pro-collagen-3 induced by the neutrophils (marked with a dash horizontal line) indicates an increase of around 10 pg/ml in the presence of 104 ug/ml Lumenato. Lumenato did not affect cell number as determined by DAPI staining (Fig 4F), suggesting that it did not induce proliferation. Of note, the cell count by DAPI was similar in the presence and absence of neutrophils since neutrophils are short lived and did not survive the overnight incubation.

**Fig 5:**
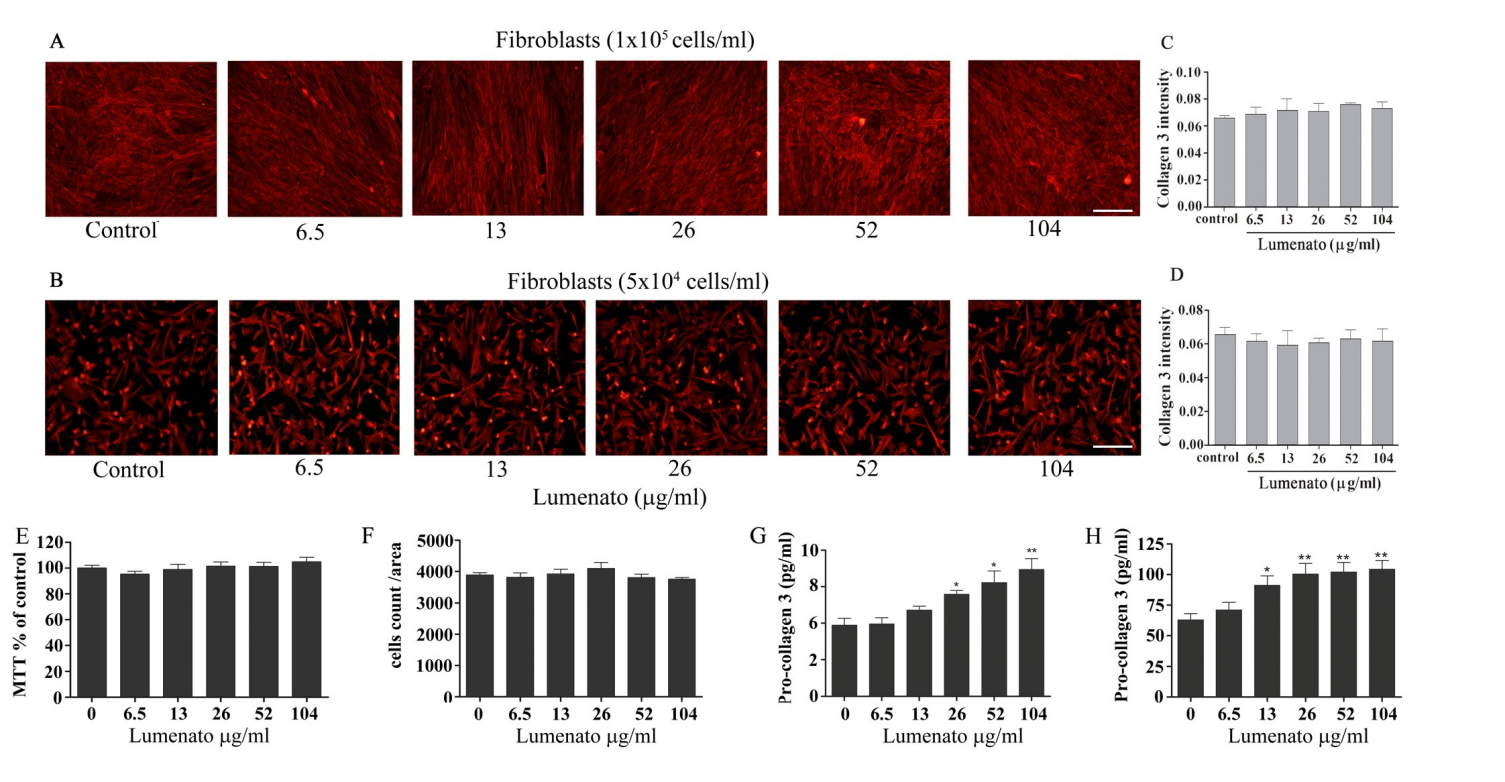
The effect of Lumenato on collagen-3 in fibroblast cultures. Addition of Lumenato in the range of 6.5–104 ug/ml to fibroblasts plated in two different concentrations did not affect collagen-3 expression, as shown by the representative immunofluorescence staining A and B, Scale bars 100 μm. The bar graphs (C and D) present the densitometry of the immunofluorescence staining of collagen-3 are the averages of three different experiments. In each experiment, five fields of each treatment were scanned. E. Addition of Lumenato did not affect cell number as measured by MTT analysis, shown mean±SEM from 5 experiments. F. Cell count measured by DAPI staining in the fields analyzed for collagen expression presented in A. G. Pro-collagen-3 in the supernatant of the co-cultures. Shown are the mean ± SEM of four different experiments each in triplicates. Significance: *p<0.05, ** p<0.01.

The effect of Lumenato on collagen-3 expression and cell number was also studied in fibroblast cultures. Lumenato was added in a range of 6.5–104 ug/ml to 1X10^5^/ml fibroblasts (the concentration used in the study) and in a lower concentration of fibroblasts that resulted in a non-confluent culture (5x10^4^/ml), enabling a better evaluation of the effect. Addition of Lumenato to the fibroblasts overnight did not affect the expression of collagen-3, as shown by a representative immunofluorescence analysis (Fig 5A and 5B) and the mean of collagen-3 densitometry of three independent experiments (Fig 5C and 5D). Addition of Lumenato to fibroblasts overnight did not affect fibroblast cell number as measured by MTT (Fig 5E) or by DAPI staining (Fig 5F), but caused a significant increase of pro-collagen-3 in a dose-dependent manner in the supernatants (Fig 5G). Of note, the elevation of pro-collagen-3 by Lumenato was similar to that detected in the co-cultures after subtracting the effect of neutrophils.

### 3.4 The protective effect of Lumenato on the release of the toxic reagents and enzymes

**Fig 6:**
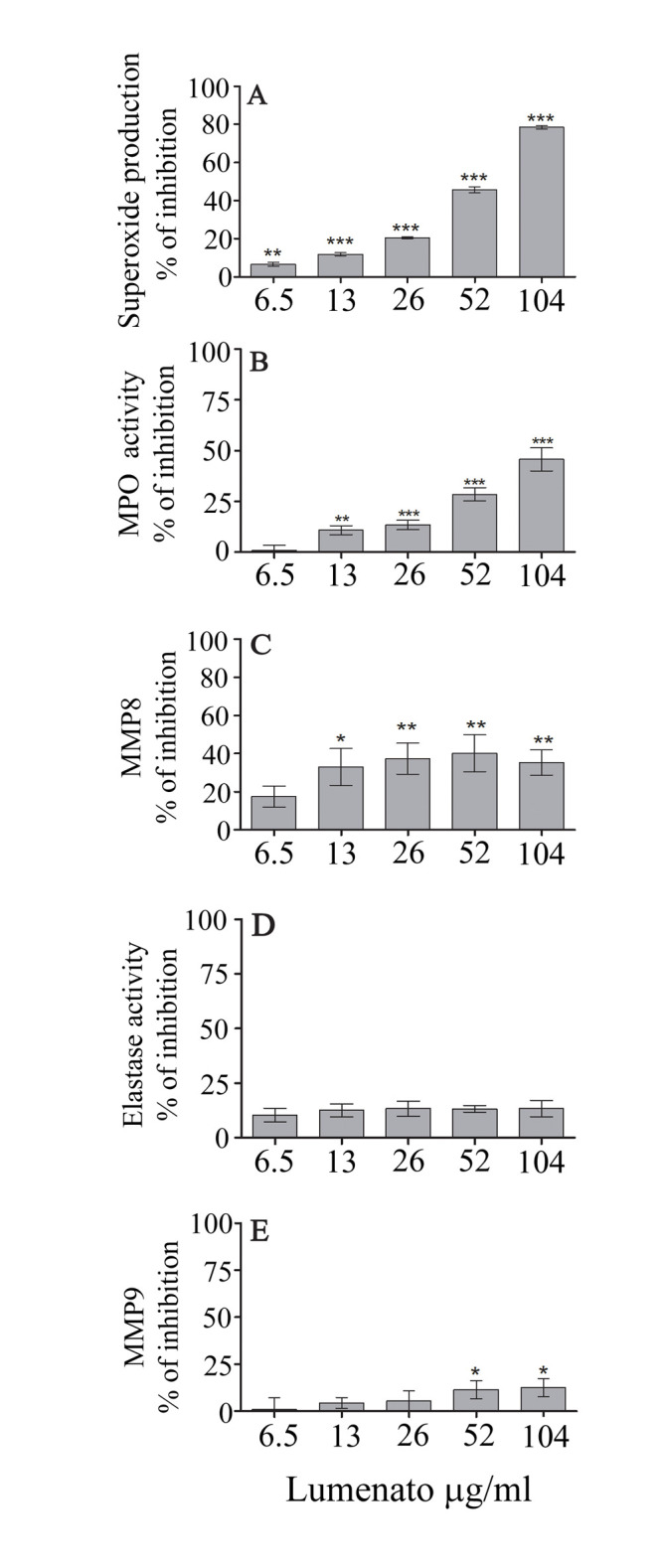
The effect of Lumenato on pro-inflammatory mediators released by activated neutrophils. Inhibition of pro-inflammatory mediators released by neutrophils activated with 100 ng/ml TNFα in the supernatant co-cultures described in Fig 4(B)–4(E). Inhibition of superoxide production (A) was measured by Amplex Red immediately after addition of the stimuli. The results are average ± SEM of three independent experiments. Significance: * p<0.05, ** p<0.01, *** p<0.001.

The effect of Lumenato on the release of the toxic reagents and enzymes from TNFα- stimulated neutrophils in the supernatant of the co-culture experiments, described in Fig 4, was measured overnight, with the exception of superoxides which were measured immediately after the addition of activated neutrophils since superoxides are not stable. The addition of TNFα–stimulated neutrophils to co-cultures caused an increase of about four times in the production of superoxides (measured by Amplex Red) from 39,500±1,326 RFU/min to 146,400±1,504 RFU/min. As shown in Fig 6A, there was a dose-dependent inhibition of superoxides by Lumenato reaching 78.5±0.9% inhibition. MPO release measured by its activity in the supernatant was doubled by TNFα- stimulated neutrophils from 8.3±1.2% to 16.6±0.25%, and its inhibition was already significant at 13 ug/ml Lumenato and gradually increased, reaching 45.8±5.6% inhibition (Fig 6B). Addition of TNFα- stimulated neutrophils to co-cultures caused an increase of about four times in the release of MMP-8 (collagenase) from 6.4±0.3 ng/ml to 28.6±1.3 ng/ml. The presence of Lumenato in the co-cultures caused a low and gradual inhibition of MMP-8 release, reaching 43.0±7.1% inhibition at 26 ug/ml which was sustained in higher concentrations of Lumenato, as presented in Fig 6C. There was a low increase of about 1.5 times of elastase release by TNFα- stimulated neutrophils in the co-cultures in comparison with the control (0.39±0.01 OD compared with 0.26±0.03 OD). Elastase release was only slightly and similarly inhibited (around 10% inhibition) by the different concentrations of Lumenato (Fig 6D). MMP-9 release measured by its activity in the supernatant was doubled by TNFα from 33.1±1.5 ng/ml to 73.4±0.8 ng/ml. Lumenato caused a gradual low inhibition of MMP-9 activity, achieving 12.5±2.1% inhibition at 104 ug/ml (Fig 6E).

## 4. Discussion

In the present study, we showed that neutrophils activated by TNFα caused collagen-3 damage in a dose-dependent manner when added to fibroblast cultures. Fibroblast-collagen-3 damage could be prevented by the addition of Lumenato to the co-cultures in a dose-dependent manner, thus, indicating its possible contribution to skin protection. Reduction of collagen-3 may result in altered organization of fibrillar collagen, thus, contributing to the wrinkled appearance of skin [8, 9]. Collagen damage can be induced by different pro-inflammatory and proteolytic enzymes from neutrophils infiltrating the skin following exposure to natural sunlight, erythemogenic dose of UVB, or solar stimulating radiation [22, 24]. Neutrophils have been shown to release various superoxides, cytokines, and proteolytic enzymes including elastase, collagenase, and gelatinase [33–35]. In order to determine the main neutrophil proinflammatory enzymes that contribute to collagen-3 damage and are inhibited by Lumenato, the activity of various enzymes was assayed in the supernatant of the co-cultures of neutrophils and fibroblasts in the presence of Lumenato at different concentrations after overnight incubation. In addition, the activity of NADPH oxidase was determined immediately in the co-cultures, as the superoxides produced are not stable. Here, we showed that although TNFα caused the release and activation of all enzymes studied, the main inhibitory effect of Lumenato was on NADPH oxidase activity and on MPO release measured by its activity. MMP-9, MMP-8, and elastase were only slightly affected by Lumenato under the same conditions. Our study shows a correlation between the dose-dependent effect of Lumenato in protecting collagen-3 damage and in inhibiting both enzymes, NADPH oxidase-producing superoxides and MPO-producing halides, suggesting that the main agents that cause collagen-3 damage are the free radicals. This suggestion is supported by the results demonstrating that collagen-3 damage measured by immunofluorescence staining could be induced by neutrophils, but not by supernatants of activated neutrophils (Fig 3), indicating that the presence of neutrophils and the short living superoxides are critical for the damage. Similar to our results, it was reported that neutrophils, but not their supernatant, enhanced collagen-1 degradation in co-cultures with corneal fibroblasts [36]. In accordance with our suggestion, recent studies reported that vina-ginsenoside R7 [37] or natural killer cell-conditioned medium [38] scavenged free radicals produced by UVB in human dermal fibroblasts and prevented collagen-1 damage. Lumenato inhibited MMP-9 release from neutrophils both alone (Fig 2) and in the co-culture experiments (Fig 6), in a dose-dependent manner in correlation with the protection of collagen damage, thus, raising the possibility that MMP-9 may also play a role in collagen-3 damage in the co-culture studies.

The combination of superoxide production and MPO release gives neutrophils a broad and unique oxidative potential. Hydrogen peroxide generated by dismutation of superoxides is utilized by MPO to oxidize halides and to generate hypochlorous acids. At physiological concentrations, hypochlorous acid was shown to be a major end-product of the neutrophil respiratory burst [39–42] with a powerful antimicrobial nature [43–45]. However, the properties that make it such a useful antimicrobial agent also place the host at considerable risk, and were implicated in the tissue injury associated with various inflammatory diseases [46], and in collagen-3 damage as shown in the present study and in accordance with the literature [36, 47]. In agreement with our results, the ingredients present in Lumenato were reported to inhibit free radicals. Vitamin E is the most abundant lipophilic antioxidant found in human skin [48] that prevents the damage induced by free radicals and reactive oxygen species [49]. Plant-derived sterols, phytosterols, structurally related to cholesterol have been shown to exert antioxidant and anti-inflammation [50, 51] in addition to reducing cholesterol synthesis. Carotenes were shown to inhibit superoxide production by NADPH oxidase [52]. The colorless carotenoids, phytoene and phytofluene, have been reported to protect from oxidation by HOCl [53]. The presence of these compounds together may have a better antioxidant effect, as shown for some of the compounds in our and other studies [52, 54–56]. Furthermore. phytoene and phytofluene are known to accumulate in the skin and absorb UV light and, thus, contribute further protection against skin damage [57].

We showed here that the effect of Lumenato in the co-cultures occurred mainly by inhibiting neutrophils' oxidative stress and not by inducing collagen synthesis, although it did increase the synthesis of collagen-3, as detected by pro-collagen-3. The concertation of collagen-3 in the fibroblasts was 0.45±0.05 ug/ml, which is in line with others [58]. Addition of neutrophils to fibroblasts caused about 25% of the collgen-3 damage (Fig 4A and 4B) around 0.11 ug/ml, and was prevented in a dose-dependent manner in the presence of Lumenato. The concentration of pro-collagen-3 was increased by Lumenato in a dose-dependent manner but only by a few pg/ml, indicating that most of the reduced damage of collagen in the presence of Lumenato is not the result of its synthesis, but protection from neutrophil damage. Had Lumenato been capable of inducing a large amount of collagen, it would have been dangerous, causing fibrosis diseases [59]. Thus, Lumenato has the potential to induce synthesis of collagen-3 at a suitable rate for maintaining skin health without inducing massive fibrogenesis.

In conclusion, activated neutrophils with TNFα-induced collagen-3 damage in fibroblast cultures is probably mediated by free radicals. The presence of Lumenato in the co-cultures prevented collagen-3 damage that correlated with the inhibition of the production of superoxides and halides in a dose-dependent manner, suggesting that Lumenato protects against collagen damage by preventing the harmful effect of neutrophils. Moreover, Lumenato induced low rates of collagen synthesis in fibroblasts that may avoid skin aging without inducing dangerous fibrinogenesis.

## Supporting information

S1 Fig Scanning the whole cover glass for collagen-3. Shown are examples of the scan of the whole cover glass for collagen-3 immunofluorescence. A. control: cover glass with fibroblasts, B. co-culture: cover glass with fibroblasts and neutrophils.
(TIF)

S2 Fig Optimal fibroblasts concentration for confluent cultures. A dose dependent fibroblast concentration (2.5X10^4^ – 2X10^5^/ml) was plated on cover glass in the 24 well plates for over night in order to define the fibroblasts concentration that results with confluent cultures but not too concentrated. Shown are representative results of immunofluorescence analysis of collagen-3. Scale bar 100 μm.
(TIF)

S3 Fig Specificity of immunofluorescence analysis of collagen-3. Representative results of immunofluorescence analysis of collagen -3 versus negative control. DAPI represents cell nuclei. Scale bar 100 μm.
(TIF)

S4 Fig Addition of TNFα did not affect collagen-3 expression. Addition of 100 ng/ml TNFα to fibroblasts for overnight did not affect collgen-3 expression as detected by immunofluorescence analysis. Scale bar 100 μm The bar graphs present the densitometry of the immunofluorescence staining of collagen-3. Shown are the means± SEM of three different experiments.
(TIF)
