# Supplementary figures and images for: Lumenato protects normal human dermal fibroblasts from neutrophil-induced collagen-3 damage in co-cultures
Source: PLoS One. 2021 Mar 17;16(3):e0248183. doi: 10.1371/journal.pone.0248183 (PMC7968672; doi:10.1371/journal.pone.0248183)

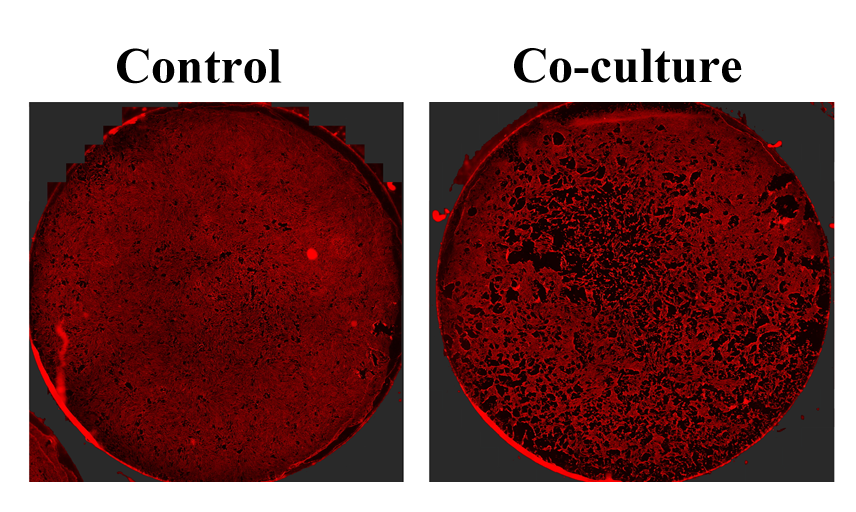

Supplement: S1 Fig — Shown are examples of the scan of the whole cover glass for collagen-3 immunofluorescence. A. control: cover glass with fibroblasts, B. co-culture: cover glass with fibroblasts and neutrophils. (TIF) [file pone.0248183.s001.tif]

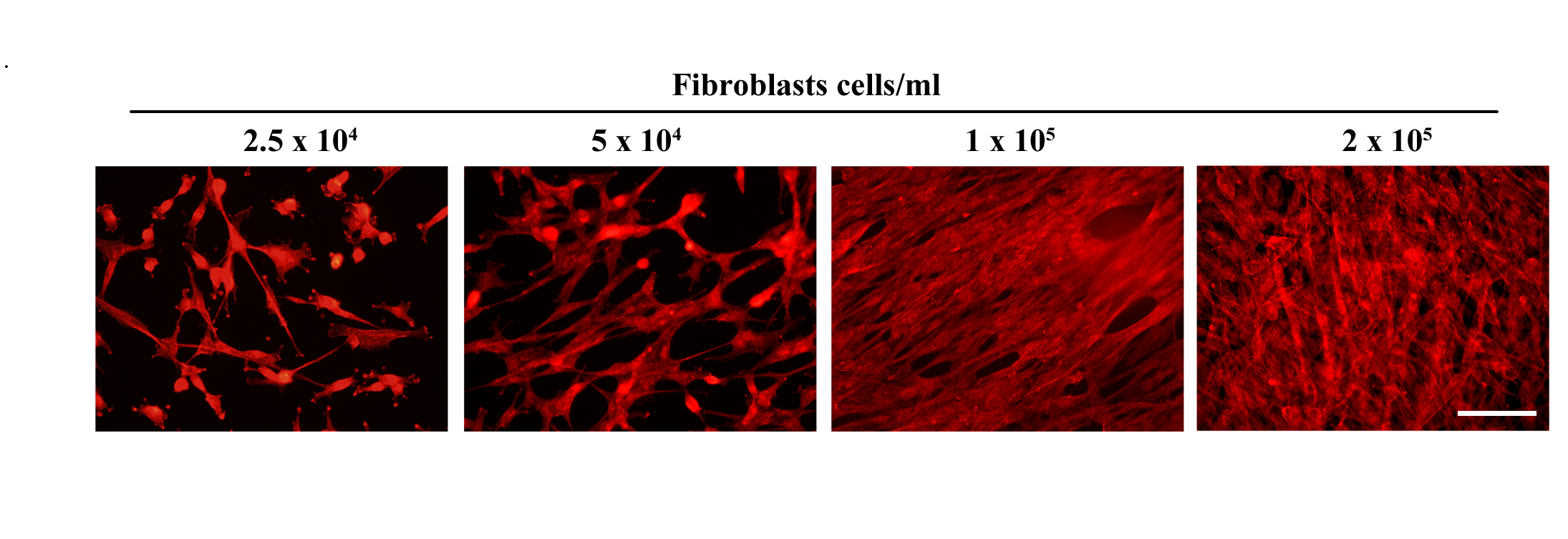

Supplement: S2 Fig — A dose dependent fibroblast concentration (2.5X104 – 2X105/ml) was plated on cover glass in the 24 well plates for over night in order to define the fibroblasts concentration that results with confluent cultures but not too concentrated. Shown are representative results of immunofluorescence analysis of collagen-3. Scale bar 100 μm. (TIF) [file pone.0248183.s002.tif]

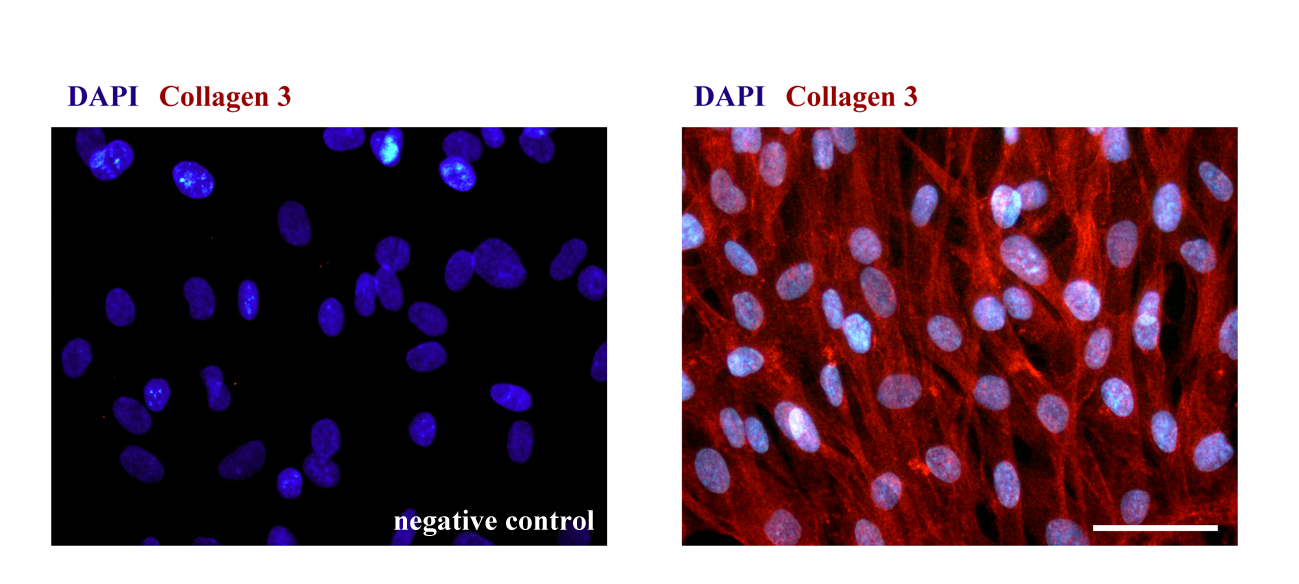

Supplement: S3 Fig — Representative results of immunofluorescence analysis of collagen -3 versus negative control. DAPI represents cell nuclei. Scale bar 100 μm. (TIF) [file pone.0248183.s003.tif]

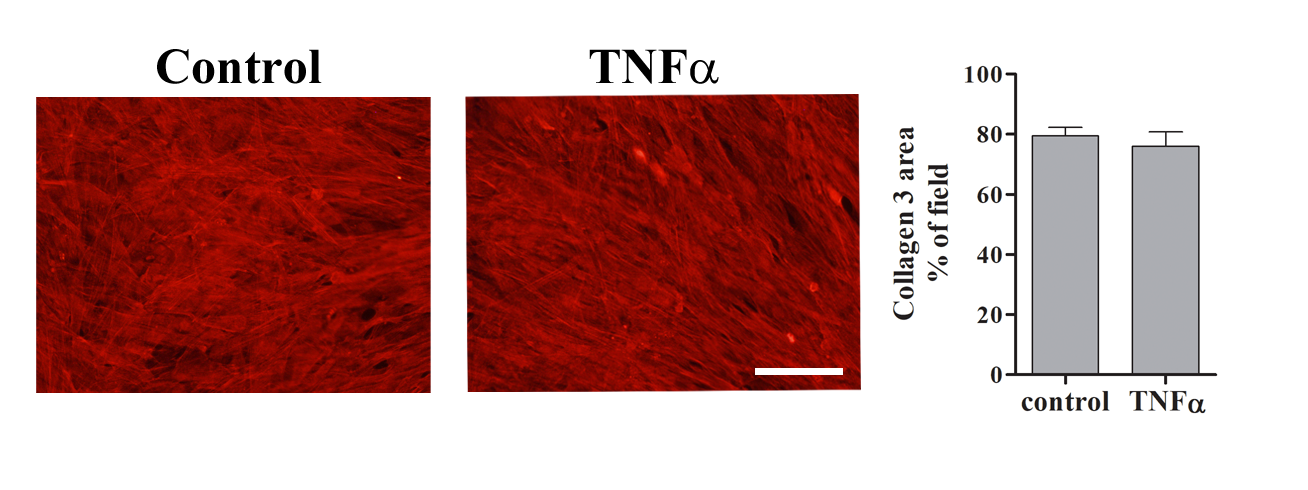

Supplement: S4 Fig — Addition of 100 ng/ml TNFα to fibroblasts for overnight did not affect collgen-3 expression as detected by immunofluorescence analysis. Scale bar 100 μm The bar graphs present the densitometry of the immunofluorescence staining of collagen-3. Shown are the means± SEM of three different experiments. (TIF) [file pone.0248183.s004.tif]
